# Laminar differences in response to simple and spectro-temporally complex sounds in the primary auditory cortex of ketamine-anesthetized gerbils

**DOI:** 10.1371/journal.pone.0182514

**Published:** 2017-08-03

**Authors:** Markus K. Schaefer, Manfred Kössl, Julio C. Hechavarría

**Affiliations:** Institute for Cell Biology and Neuroscience, AK Neurobiology and Biosensorics, Goethe University, Frankfurt/Main, Germany; Universidad de Salamanca, SPAIN

## Abstract

In mammals, acoustic communication plays an important role during social behaviors. Despite their ethological relevance, the mechanisms by which the auditory cortex represents different communication call properties remain elusive. Recent studies have pointed out that communication-sound encoding could be based on discharge patterns of neuronal populations. Following this idea, we investigated whether the activity of local neuronal networks, such as those occurring within individual cortical columns, is sufficient for distinguishing between sounds that differed in their spectro-temporal properties. To accomplish this aim, we analyzed simple pure-tone and complex communication call elicited multi-unit activity (MUA) as well as local field potentials (LFP), and current source density (CSD) waveforms at the single-layer and columnar level from the primary auditory cortex of anesthetized Mongolian gerbils. Multi-dimensional scaling analysis was used to evaluate the degree of “call-specificity” in the evoked activity. The results showed that whole laminar profiles segregated 1.8-2.6 times better across calls than single-layer activity. Also, laminar LFP and CSD profiles segregated better than MUA profiles. Significant differences between CSD profiles evoked by different sounds were more pronounced at mid and late latencies in the granular and infragranular layers and these differences were based on the absence and/or presence of current sinks and on sink timing. The stimulus-specific activity patterns observed within cortical columns suggests that the joint activity of local cortical populations (as local as single columns) could indeed be important for encoding sounds that differ in their acoustic attributes.

## Introduction

Communication sounds play a fundamental role in intra-species interactions. The neuronal representation of communication sounds has been investigated in a number of mammalian species, including the squirrel monkey, *Saimiri sciureus* [1], the rhesus monkey, *Macaca mulatta* [2–5], the long-tailed macaque *Macaca fascicularis* [5–6], the marmoset, *Callithrix jacchus* [7–9], the domestic cat, *Felis domestica* [8, 10–11], the mustached bat, *Pteronus parnellii* [4, 12], the short-tailed bat, *Carollia perspicillata* [13–15] and the house mouse, *Mus muscus* [16–17], among other species. All the aforementioned studies agree in that the processing of communication sounds is different from that of more “simplistic” sounds, such as pure-tones or broadband noise [18–19]. This presumably is due to the complex spectro-temporal structure of communication sounds, consisting of combinations of constant frequency, frequency-modulated (FM), and noise-burst (NB) components that often present a harmonic structure [4].

The discrimination of communication sounds is very similar between nonhuman mammals and humans indicating the existence of general auditory processing mechanisms shared across species [20–25]. Animal models have provided us with valuable information about the basic neurophysiologic mechanisms that could underlie human communication sound perception. Experiments on animals revealed that the time-varying frequency spectra of complex sounds are efficiently separated into narrow bands at the level of the inner ear [2, 26]. Research on the auditory nerve provided valuable information about the auditory encoding at the periphery [27–29]. The auditory information generated at the lower auditory stations converges in the inferior colliculus [30–31]. At this station, neurons with spectral integration properties emerge for the first time [32–33]. This important mechanism for the integration of information across different frequencies generates a selective response to communication calls [34]. Although the IC has been termed as a prime site to examine neural processing of complex sounds, how information about the tonal composition is reassembled and how this information gives rise to perceptual and cognitive performance can only be answered in higher functional areas, such as the cerebral cortex.

Theories from the 1970s proposed that specific “call detectors” were responsible for the encoding of species-specific vocalizations [1, 35–36]. Although certain neurons could be described as “call detectors” [35, 37], it has been shown that the majority of central auditory neurons respond to more than one call, or to various features of the calls [10, 12, 38–39]. Later studies proposed models for encoding communication sounds that were based on the activity of neuronal populations, rather than in single units [4, 7, 40]. The mechanisms by which the brain encodes communication sounds have been widely studied in the last decades. Yet, to date, there is no clear consensus on whether communication sound encoding depends on single neuron or on population activity across multiple neurons (or both). One possible hypothesis is that the activity of local neuronal assemblies, such as those occurring in cortical columns, could provide sufficient information for distinguishing between different spectro-temporal call properties. However, this distinction could be influenced by the neural signal type that is analyzed. For example, at the single-layer level Medvedev and Kanwal [12] could show that the neuronal spiking activity was less informative about the properties of the sounds than the local field potentials (LFPs). It has been discussed that the differences between spike and LFP responses are a direct consequence of the different origin of those two signal types [12, 41]. Spiking activity reflects the processed neuronal output around the near field of the electrode, while LFPs entail the summed potentials (inhibitory and excitatory) of a larger region including the activity from several columns and even activity from extra-cortical areas [42–43]. From simultaneously recorded LFP signals another signal type can be calculated, the so-called current source density (CSD).

CSD patterns are based on the 2nd spatial derivative of the LFPs recorded across several channels. It has been said that CSD patterns can provide insights into the sound encoding mechanisms by exactly localizing the sound-specific information flow between cortical layers [44–45].

In the present study, we analyzed the columnar activity in the primary auditory cortex (AI) of gerbils (*Meriones unguiculatus*) obtained in response to both pure tones and communication calls reaching into the ultrasonic range. Ultrasonic vocalization is an essential part of social behavior in rodents, influencing their social interactions, reproductive success, and survival [46–48]. Although the relevance of ultrasonic calls is well documented, how these calls are represented at the cortical level, and especially along the cortical columns, remains poorly understood. Our aim was to determine whether spectro-temporally different sound attributes lead to different cortical columnar activation patterns. We analyzed pure-tone and communication-sound elicited multi-unit activity (MUA), local field potential (LFP), and current source density (CSD) waveforms at the single-layer and columnar level in the AI of anesthetized Mongolian gerbils. Our results show that complex communication sounds elicit unique laminar MUA, LFP, and CSD profiles that differ significantly across acoustic stimuli. Moreover, we show that the stimulus-evoked activity is more “informative” in whole laminar profiles than in individual layers and more “informative” in LFP and CSD profiles than in MUA profiles.

## Results

**Fig 1:**
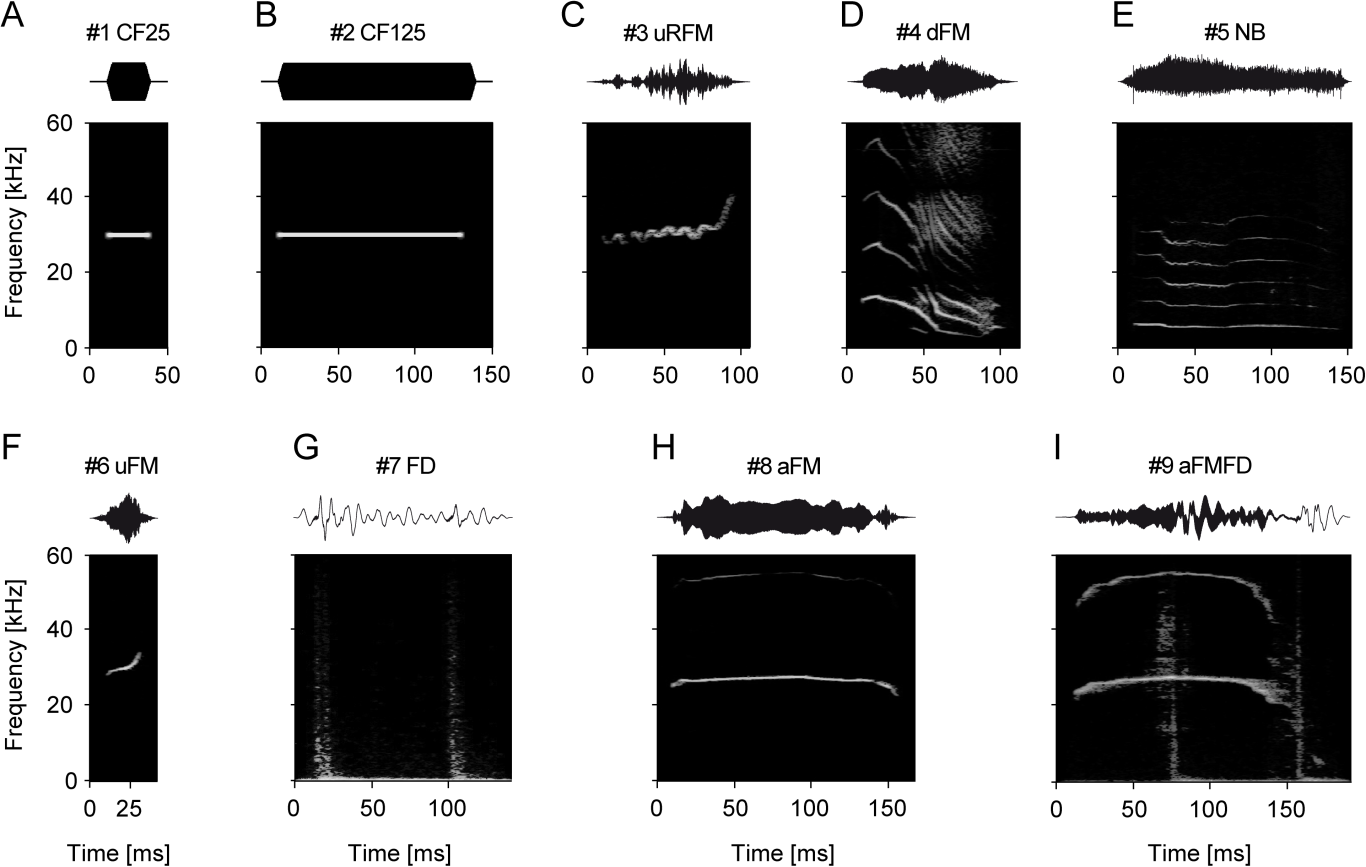
Pure-tones and communication sounds of Mongolian gerbils. Panels show the oscillograms (top) and the corresponding spectrograms (bottom) of each stimulus used in the present study. In addition to the pure-tone controls of 25 and 125 ms duration [#1 CF25 (A) and #2 CF125 (B)], multiharmonic sounds emitted during mating and discomfort [#3 uRFM (C)], isolation [#4 dFM (D)], agonistic interaction [#5 NB (E)], non-conflict mating [#6 uFM (F)], and alarm behavior [#7 FD (G), #8 aFM (H), and #9 aFMFD (I)] were presented. All communication sounds were recorded from the same breeding colony which was used for the experiments. The set of sounds was chosen to cover the species-specific variety of different spectro-temporal properties.

**Fig 2:**
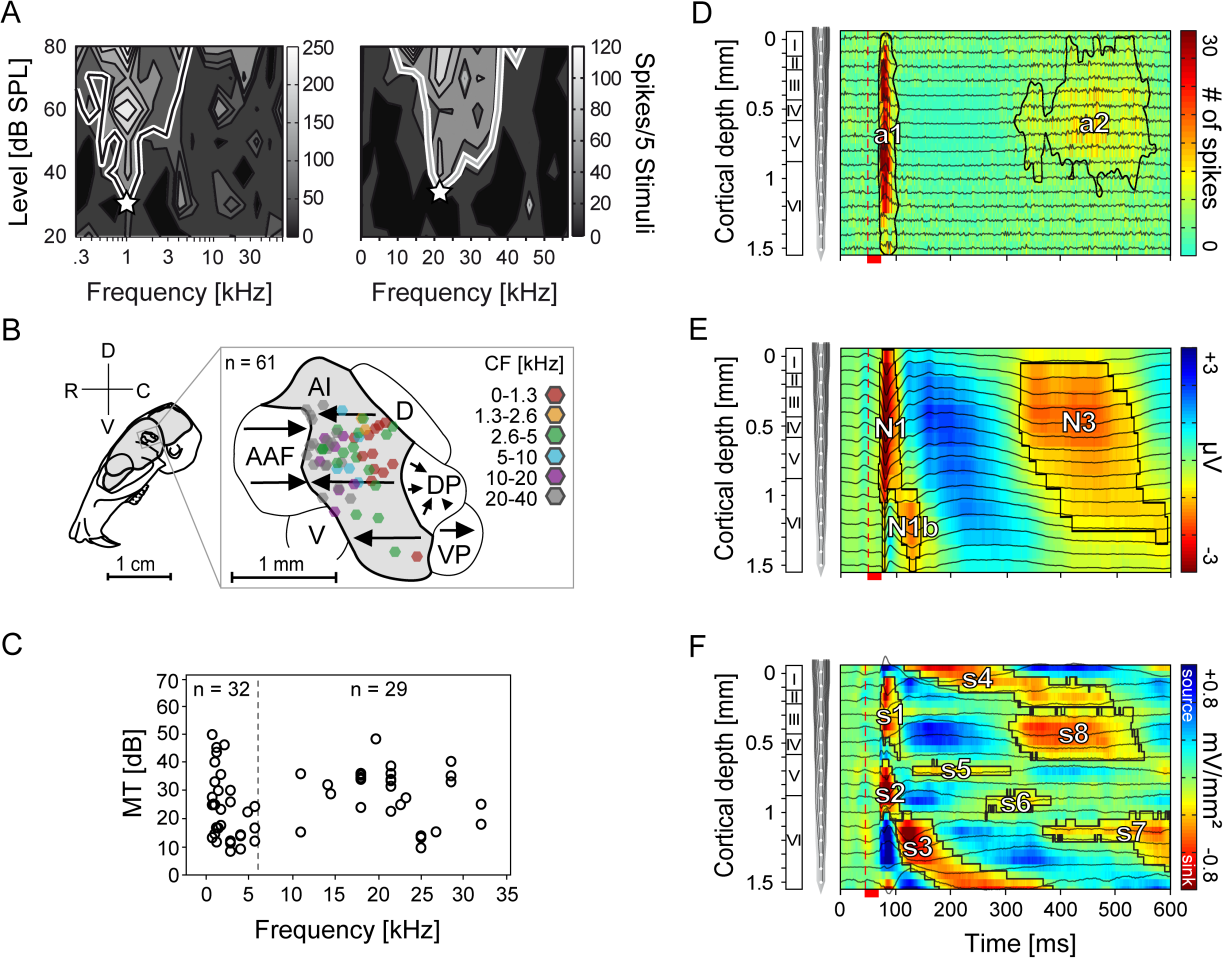
Frequency tuning and cortical location of neurons and representative laminar MUA, LFP, and CSD profiles. (A) The left and right graphic shows two representative MUA-based tuning curves obtained from neurons in layer IV of the same animal. The white asterisks represent the CF located at 1 and 21 kHz, respectively. (B) Figure shows the color-coded locations of the recording sites within the schematic representation of the parcellation of the auditory cortex [(AAF (anterior auditory field), AI (primary auditory cortex), D (dorsal field), DP (dorsoposterior field), V (ventral field), VP (ventroposterior field)]. Arrows indicate the tonotopic organized cortical areas. All laminar tracks (n = 61) were recorded within the AI and marked as hexagons whose color represents the characteristic frequency (CF). Adapted from [51–52] and reprinted from the open access journal [49] under a CC BY license. (C) The figure displays the median characteristic frequency (CF) against the median minimum threshold (MT) of tuning curves interpolated from neuronal spike responses of layers III-VIa. The high/low CF border was defined at 6 kHz (dashed line). (D, E) Graphics show single laminar profiles (averaged over 50 trials) of one cortical site constructed from the neuronal activity filtered at 300-4500 Hz for MUA (D) and 0.1-300 Hz for LFP (E) recorded from the same location tuned to 1.4 kHz with a linear-array multicontact electrode covering all six cortical layers. Neuronal recordings were obtained simultaneously at 16 depths (black traces) at an interchannel distance of 100 μm and represented as a colormap in the background. (F) The single CSD profile was calculated from the second spatial derivative of the local field potentials (E). Red colored current sinks labeled as s1 to s8 are classically interpreted as net inward transmembrane currents and current sources (blue) as net outward currents. Contours enclosing the neuronal activity were calculated at 6 spikes (MUA, a1 and a2) and 20% (LFP, N1, N1b, and N3) or 10% (CSD, s1-s8) of the maximum negative amplitude of the respective profile. The vertical dashed line marks the beginning of pure-tone stimulation (1.4 kHz; 80 dB SPL). Red scale bar represents the stimulus duration of 25 ms.

A precise quantification of the layer-dependent responses to sounds differing in their spectro-temporal properties could shed light onto communication-sound encoding principles. The neuronal responses to pure-tones (Fig 1A and 1B) and seven complex communication sounds (see stimuli in Fig 1C–1I), which were selected to cover a variety of acoustic attributes and behavioral situations, were compared. The carrier frequency of the simple pure-tones of different durations (25 and 125 ms) was set at the CF of each cortical column obtained from neuronal tuning curves (see example in Fig 2A). The data were sampled in anesthetized animals to obtain a measure of basic columnar network activity without active modulation, e.g. by attentional feedback. A description of the communication sounds used as stimuli can be found in the Materials and Methods section. Responses to pure tones were assumed to be more simplistic in their structure in comparison to responses evoked by complex communication calls. Sound-evoked MUA, LFP, and CSD activity were compared at the single layer and laminar profile level to gather data on the cortical operation principles.

### 2.1. Stimulus-specific characteristics of laminar MUA, LFP, and CSD profiles

We compared the stimulus-evoked activity obtained using three different neural signal types (MUA, LFP, and CSD). For the laminar analysis of neural activity in AI, the time courses of neural signals obtained in 61 cortical sites (colored hexagons, Fig 2B) were studied using laminar probes (16 channels). Across cortical sites, neurons had a homogeneous frequency tuning distribution (Fig 2C). Neurons of 32 penetration points were tuned to low frequencies (CF < 6 kHz) whereas neurons of 29 penetration points were tuned to high frequencies (CF > 6 kHz). The neuronal activity obtained from each of the 16 electrode channels of each recording site was linearly merged in order to represent the location and extent of the activity within cortical columns. Representative examples of one cortical site of these so-called laminar profiles are shown in Fig 2D–2F for each of the three neural signal types studied (i.e. MUA, LFP, and CSD).

**Fig 3:**
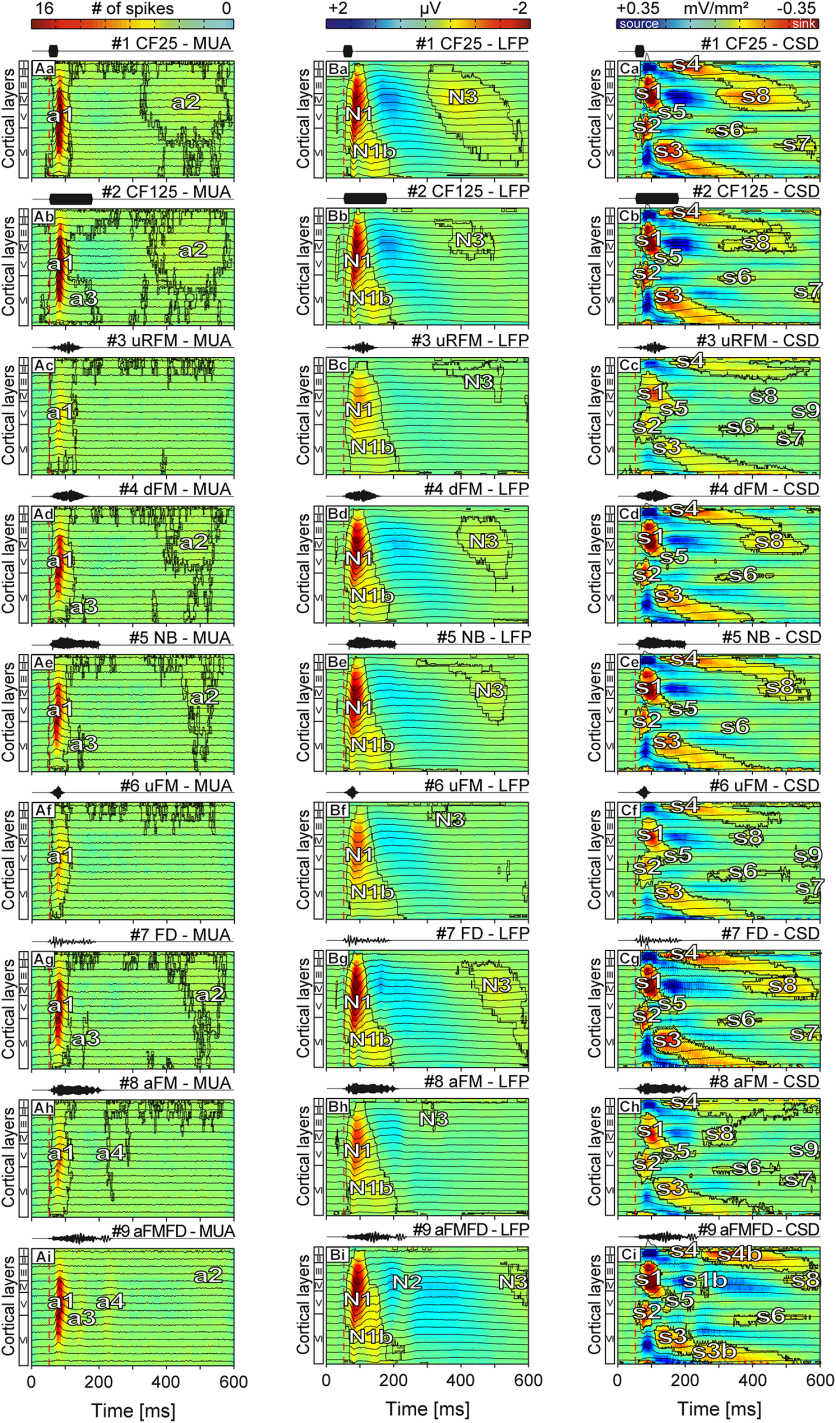
Median laminar MUA, LFP and CSD profiles of stimulus-evoked recordings. Each point in the profiles represents the median of the averaged (n = 61) depth-adjusted MUA (Aa-Ai), LFP (Ba-Bi), and CSD (Ca-Ci) in the response to two pure tones (rows 1-2) and 7 different spectro-temporally complex sounds (rows 3-9). Contours enclosing the neuronal activity (a1-a4) in the MUA profiles were calculated at a threshold of 4 spikes whereas contours in the LFP (N1-N3) and CSD profiles (s1-s9) were calculated at a threshold of 5% of the maximum negative amplitude of the respective LFP or CSD profile. While the overall laminar structure remains similar most of the MUA, LFP, and CSD profiles show characteristic stimulus-specific differences. Most of the activity responsible for these differences is found at > 100 ms post stimulus. A stimulus frequency dependent dissimilarity in the MUA strength and profile structure is prominent in (Ac), (Af), and (Ah). The vertical dashed line marks the beginning of stimulation. Corresponding oscillograms of stimuli are shown above the profiles.

In Fig 3Aa–3Ai the median laminar profiles of cortical spiking activity in response to each of the tested stimuli are represented. The interquartile range of the respective median columnar activity profiles can be found in the Supplementary section (S1 Fig). Note that we decided to pool together the MUA, LFP, and CSD activity profiles obtained in columns tuned to low and high frequencies (S2 Fig), since the LFP, CSD, and most of the MUA temporal courses correlated quite well with one another (R > 0.59, S3 Fig). In general, the spiking profiles (Fig 3Aa–3Ai) were composed of a short (~60 ms in duration) initial phasic activity (denoted as a1) that was strongest in layers II-VIa. The contoured weak late activity denoted as “a2” in the figure (~100-250 ms in duration) was not present in some profiles (Fig 3Ac, 3Af and 3Ah) at the applied contouring threshold (that is, 4 spikes). In most of the remaining profiles (Fig 3Ab, 3Ad, 3Ae, 3Ag and 3Ai) an additional activity (a3) limited to infragranular layers could be found, which was vertically extended and temporally close to the activity “a1” in response to the aFMFD stimulus (Fig 3Ai).

The stimulus-specific median LFP profiles were composed of three negativities colored in red (N1-N3) and one large positivity colored in blue located between N1/N1b and N3 (Fig 3Ba–3Bi). The N1 activity, which was located between layers II-VIa, lasted about 1.5 times longer than the a1 activity in the corresponding MUA responses (i.e. ^~^90 ms (N1) versus ~60 ms (a1), see Fig 3Aa–3Ai). The N1b activity was restricted to layer VI and followed the N1 activity with a delay of ~25 ms. The late N3 potential had relatively small amplitudes (~10% of the N1 amplitude) and mainly peaked in layer IV.

Median CSD profiles in the right column (Fig 3Ca–3Ci), that were calculated on the basis of the LFPs (see Materials and Methods), displayed a complex structure classically consisting of eight contoured sinks (s1-s8, colored in red) that appeared temporally separated by return currents (sources) [49]. The separated initial activity areas (s1 and s2), that represent the putative cochleotopic input from the ventral and medial division of the medial geniculate body [50], were located in layers III-IV and Vb-VIa, respectively. The remaining sinks were restricted to one specific layer and had smaller amplitudes in comparison to the initial sinks.

Overall, laminar MUA, LFP, and CSD profiles differed in their complexity, and in the onset latency and duration of the areas that indicate neural activity (red areas in the profiles, see Fig 3). LFP and CSD responses, but not MUA responses, to short pure tones (i.e. 25 ms duration, Fig 3Ba and 3Ca) and to long pure tones (i.e. 125 ms duration, Fig 3Bb and 3Cb) showed a relatively strong amplitude difference in the contoured N3 (LFP profiles) and s8 (CSD profiles). Also, high frequency sounds such as stimuli #3, #6, and #8 elicited weak MUA, LFP, and CSD activity strengths when compared to responses evoked by the remaining stimuli. The latter was probably due to the relatively weak activity of low frequency tuned neurons in response to high frequency sounds (S2 Fig). Qualitatively, additional structural differences across LFP and CSD profiles evoked by different sounds appeared in the form of the presence/absence of late sinks [i.e. s7 and s9 (CSD profiles)], as well as in the duration of N3 and N1 depolarizations (LFP profiles). Interestingly, all median CSD profiles elicited by the three stimuli consisting solely of high frequencies (i.e. stimuli #3, #6, and #8, Fig 3Cc, 3Cf and 3Ch) showed a late sink s9 which was also found in a previous study in profiles evoked by pure-tones one octave above the CF [49].

The clearest stimulus-specific differences between MUAs, LFPs, and CSDs were found when comparing the activity profiles evoked by stimuli #8 and #9 with those evoked by the remaining stimuli. For example, MUA profiles obtained in response to stimuli #8 and #9 showed an additional weak neuronal activity (a4) representing either a response to the stimulus offset or a response to the 2nd drumming event (Fig 3Ah and 3Ai). In the LFP domain, stimulus #9 (Fig 3Bi) evoked a weak negativity labeled as N2 (non-contoured); while in the CSD profile sinks appeared (i.e. s1b, s3b, and s4b) that were not observed in response to the remaining sounds (Fig 3Ci). Additionally, the area N3 (LFP) and sink s8 (CSD) were temporally shifted (> 450 ms post stimulus) when comparing responses to stimuli #8 and #9 with profiles evoked by the remaining stimuli. Also, in response to stimuli #9 sink s7 was found to be completely missing (Fig 3Ci). The weak negative potential N3 and the sink s8 elicited by stimulus #8 (Fig 3Bh and 3Ch) were both characterized by short onset latencies (~220 ms post stimulus) and a decrease in the amplitude of the preceding negativity or source in comparison to the profiles elicited by the remaining stimuli. Additionally, the sink s4 in the CSD profile had a longer duration in comparison to corresponding sinks in Fig 3Ca, 3Cb and 3Cd–3Cg.

In conclusion, based on MUA, LFP, and CSD profiles it is possible, at least on a qualitative basis, to differentiate between sounds that differ in their spectro-temporal properties and LFP and CSD activation profiles show the strongest stimulus-dependent structural differences.

### 2.2. Statistical analysis of stimulus-induced changes in the laminar profiles

The observed structural differences described in the preceding section are based on qualitative observations. A statistical comparison between the stimulus-specific response profiles is necessary to validate the observed stimulus-specific laminar profile differences. For this, a parametric repeated measures analysis of variance in combination with a false discovery rate post-hoc test was used [53]. The averaged (50 repetitions) measurement points of the two-dimensional response profiles (resolution: 16 channels located along the cortical depth x 600 time points) recorded from all 61 cortical sites were non-independently compared using repeated measures (F(df1, df2) = 8235, 528564). For simplicity, the MUA, LFP, and CSD profiles elicited by the long pure-tone stimulus (#2 CF125, duration = 125 ms) were tested against the respective measurement points of all remaining profiles. In other words, this comparison would tell us how different a given response profile is, when compared to the response obtained when playing a long artificial pure-tone. Note that the duration of the control pure tone (that is, 125 ms) corresponds to the mean duration of stimuli #3-#5 and #7-#9. The statistical analysis between all remaining profile combinations can be found in the Supplementary section (S4 Fig). The MUA profiles, which were calculated with a bin size of 2 ms, were interpolated to match the number of points of LFP and CSD profiles.

By statistically comparing the laminar MUA, LFP and CSD profiles evoked by stimulus #2 with those evoked by the remaining stimuli we wanted to seek answer to the following questions: (I) Which response signal type, based on the respective amount of significantly different columnar activation areas, possesses the most information related to stimulus encoding?; and: (II) Is the “call-specificity” of profiles, based on the stimulus-specific structure of significantly different response areas high enough for encoding different spectro-temporal call properties?

**Fig 4:**
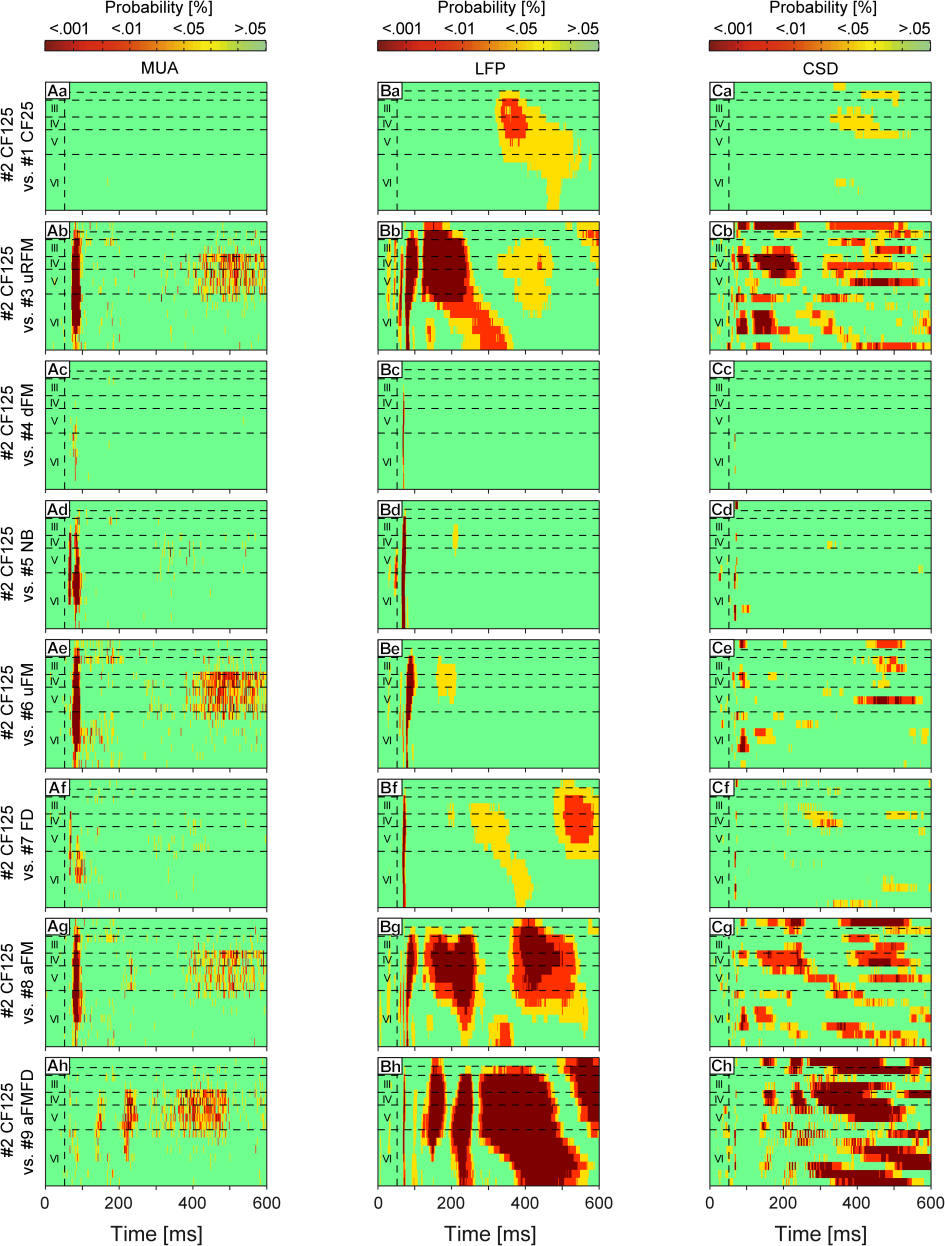
Statistical comparison between laminar MUA, LFP, and CSD profiles. A parametric repeated measures ANOVA in combination with a false discovery rate post-hoc test was applied for 61 measurement points at each of the 600 time points and all 16 channels of the MUAs (Aa-Ah); the LFPs (Ba-Bh), and the CSDs (Ca-Ch). The profile evoked by stimulus #2 CF125 was set as control and tested against all remaining profiles. The points in the profiles represent the probability values (green: p ≥ 0.05; yellow: p < 0.05; red: p < 0.01; dark red: p < 0.001). Horizontal dashed lines indicate layer borders while the vertical dashed line marks the beginning of stimulation.

The results of the statistical analysis are displayed in the form of laminar profiles in which the color code indicates the level of significance (Fig 4; green: p ≥ 0.05; yellow: p < 0.05; red: p < 0.01; dark red: p < 0.001) obtained when comparing each time point (n = 61) with the respective time points of the control profiles (see preceding text). Comparing the MUA profiles evoked by high frequency stimuli #3, #6, and #8 (Fig 4Ab, 4Ae and 4Ag) with that obtained in response to stimuli #2 (that is, the control stimulus: a pure-tone whose frequency was equal to the CF of the column) revealed significant differences in the initial phasic response (a1) and the late activity (a2). These differences were likely caused by the fact that in 52% of the cortical sites studied were tuned to low frequencies (see Fig 2C; CFs < 6 kHz). Note that low frequency tuned neurons will only respond weakly to communication sounds in which most of the energy is located at high frequencies (as it is the case of sounds #3, #6, and #8; S2 Fig). The MUA profile evoked by stimulus #5 (Fig 4Ad) showed significant differences when compared against the control stimulus, and those differences were mainly restricted to the initial phasic response (a1). Some measurement points located in the areas a1-a3 elicited by stimuli #4 and #7 (Fig 4Ac and 4Af) also differed significantly from the response to the long pure tone, indicating minor stimulus-specific changes across MUA profiles. The profile elicited by stimulus #9 (Fig 4Ah) differed considerably regarding the activities a2-a4 in comparison to the profile elicited by stimulus #2.

For an objective description of the dissimilarity level between profiles we calculated the magnitude of significance index (abbreviated “MS”) describing the percentage of points of each profile that was significantly different to the respective response template (that is, the response to stimulus #2 CF125) with p < 0.05. Note that this index contains data from all significant areas including negativities/sinks and positivities/sources in the LFP and CSD profiles, respectively. MUA profiles evoked by stimuli #1 (MS = 0.02%), #4 (MS = 0.6%), #5 (MS = 4.2%), and #7 (MS = 3.2%) showed small MS values, whereas profiles evoked by stimuli #3 (MS = 14.1%), #6 (MS = 18.2%), #8 (MS = 13%), and #9 (MS = 14.3%) showed relatively high MS values.

In contrast to the MUA profiles, the LFP profiles (Fig 4Ba–4Bh) contained multiple areas which differed significantly from the respective areas of the profile elicited by the pure-tone stimulus #2. LFP profiles evoked by stimuli #4 (MS = 0.5%), #5 (MS = 2.9%), and #6 (MS = 5.8%) showed small significant differences when compared to the profile evoked by stimulus #2, and those differences were located mainly in the initial part of N1 (Fig 4Bc–4Be). Higher MS values could be found in profiles evoked by stimuli #1 (MS = 18.6%) and #7 (MS = 21.7%) (Fig 4Ba and 4Bf). These profiles showed significant differences in the areas where the depolarization N3 and the preceding repolarization field were located. About half of the area in the profiles evoked by stimuli #3 (MS = 43.8%), #8 (MS = 49.3%), and #9 (MS = 59.7%) differed significantly from the profile evoked by the control pure-tone (Fig 4Bb, 4Bg and 4Bh). The negativities N1, N2, and N3 and the repolarization field between N1 and N3 were the main targets of these significant differences. All profiles obtained with complex sounds (Fig 4Bb–4Bh) had in common significant differences with the control profile in the first 10 ms of the field N1. This could have been caused by the steeper rise of stimulus energy in the pure-tone stimuli compared to the complex sounds (see stimuli in Fig 1).

The statistical analysis of CSD profiles (Fig 4Ca–4Ch) revealed significant stimulus-specific differences covering similar areas in comparison to LFP profiles. CSD profiles evoked by stimuli #4 (MS = 0.1%) and #5 (MS = 1.4%) were very similar in comparison to the control profile as indicated by the absence of significant differences (Fig 4Cc and 4Cd). The profiles evoked by stimuli #1 (MS = 6.3%), #6 (MS = 11.5%), and #7 (MS = 6.3%) showed some significant differences which were mainly located around sink s8, its preceding source in layer IV, sink s7, and the sources preceding sink s3 and following sink s5 (Fig 4Ca, 4Ce and 4Cf). The highest MS values could be found in the profiles evoked by stimuli #3 (MS = 42.3%), #8 (MS = 35.7%), and #9 (MS = 45.3%) (Fig 4Cb, 4Cg and 4Ch), in accordance to what was found in the LFP profiles (see preceding text). The presence of sink s9 located in layer V also rendered significant differences when comparing against the control profile (Fig 4Cb, 4Ce and 4Cg). In conclusion, the MUA, LFP, and CSD profiles evoked by all stimuli but #4 and #5 showed stimulus-specific significant differences in comparison to the profile evoked by stimulus #2. These differences in response profiles were highest in LFP and CSD data.

### 2.3. Multi-dimensional scaling of stimulus-specific responses

With the statistical analysis (see Fig 4) we could exactly localize the significant differences between the profiles evoked by stimulus #2 (control) and the remaining stimuli. However, statistical analysis cannot resolve the degree of discriminability across stimuli based on layer-specific waveforms and laminar profiles. To evaluate the degree of dissimilarity (or similarity) in the layer-specific response waveforms and laminar profiles evoked by different stimuli, we conducted a multi-dimensional scaling (MDS) analysis. This method reduces the high-dimensional data into a low-dimensional target space in which the data is reconstructed as best as possible reflecting the maximized goodness-of-fit [54]. The “distances” between pairs of items represent their degree of dissimilarity, and the ability to separate between items is given by the “stress” factor [54].

**Fig 5:**
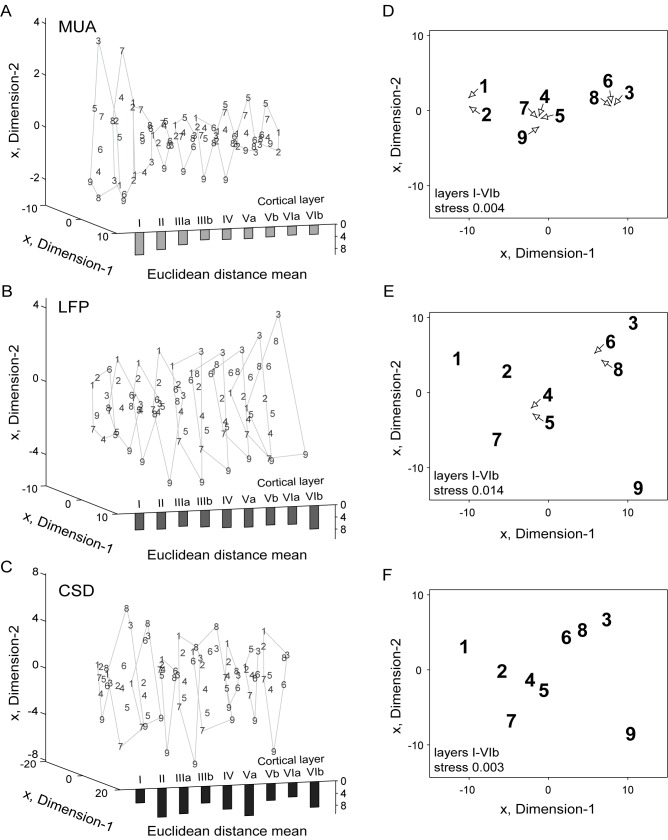
Multi-dimensional scaling (MDS) of averaged stimulus-evoked responses. Graphics display the two-dimensional MDS of the averaged responses of each layer (A-C) and the median MUA (D), LFP (E), and CSD (F) profiles of all layers. In (A-C) layers are indicated with Roman numerals. (A-F) Arabic numbers represent the stimuli eliciting the respective waveforms/profiles. (D-F) Arrows point to the actual position of profiles. Solid lines in the left graphics (A-C) indicate the outer borders of the layer-wise MDS. The mean of all Euclidean distances between the waveforms elicited by stimuli #1-#9 are displayed as bars at the bottom for each layer. The averaged waveforms or profiles were normalized to the respective stimulus-independent maximum value before calculation. The degree of dissimilarity between LFP and CSD waveforms/profiles is increased in comparison to the MUA. The whole laminar profiles segregate 2.3 (MUA), 2.6 (LFP), or 1.8 (CSD) times better across different calls than single-layer activity.

In Fig 5 we analyzed the MDS at the two-dimensional level which is equivalent to the usage of the first two principal components capturing the largest possible variances [98.6% (MUA); 98.1% (LFP); 97% (CSD)] of the profiles [55]. The MDS of post-stimulus evoked MUA, LFP, and CSD responses averaged over all recording sites (n = 61), which are marked as respective Arabic numbers in the abstract representational space, was analyzed for each layer (or sub-layer) separately (Fig 5A–5C, resolution: 1 channel x 2750 time points, sampling rate: 20 kHz) and conjointly (Fig 5D–5F, resolution: 16 channels x 2750 time points, sampling rate: 20 kHz). We normalized all MUA, LFP, and CSD waveforms to the respective stimulus-independent maximum value to eliminate entity-dependent magnitude differences between the three types of signals studied (that is, MUA, LFP, and CSD). As for the previous analysis, MUA profiles, which were calculated with a bin size of 2 ms, were interpolated to match the number of points of LFP and CSD profiles.

As indicated by the small Euclidean distances [4.2 ± 1.6 (mean ± SD)], the single-layer MUA waveforms (Fig 5A) segregated moderately, showing relatively small differences between the waveforms evoked by the 9 different stimuli. For MUA signals, the segregation performance (taken as the mean Euclidean distance) between stimulus-driven waveforms gradually decreased from layer I to layer IIIb and remained relatively similar between layers IIIb-VIb. The LFP waveforms were, in contrast to the MUA waveforms, arranged in the two-dimensional space in a relatively constant configuration throughout layers IV-VIb (Fig 5B). However, the Euclidean distances were larger [5.9 ± 0.8 (mean ± SD)]. CSD waveforms showed the largest Euclidean distances [7.3 ± 2.2 (mean ± SD)] which were highest for layer Va [10.3 ± 4.1 (mean ± SD)] (Fig 5C).

Applying the MDS on whole laminar profiles resulted in an increase of segregation performance in all three types of neuronal activity by factor 2.3 (MUA), 2.6 (LFP), or 1.8 (CSD), measured as the mean Euclidean distances between waveforms/profiles (Fig 5D–5F). The clustered MUA profiles evoked by the high frequency stimuli #3, #6, and #8 (Fig 5D) were located at a large Euclidean distance from the remaining profiles, which is in accordance to the results described in preceding sections (see Figs 3 and 2). The profiles elicited by stimuli #4, #5, #7, and #9 were clustered together and were significantly separated from the profiles elicited by stimuli #1 and #2. The two-dimensional configuration of stimulus-induced MUA profiles reflected the differences between the profiles with a high reliability, as indicated by the low stress factor of 0.004 [note that stress factors < 0.1 are considered as good [56]].

In laminar (as opposed to single layer) LFP profiles the stress was equal to 0.014, indicating consistent dissimilarities in laminar LFP data (Fig 5E). LFP profiles evoked by stimuli #2, #4, #5, and #7 showed relatively small Euclidean distances between each other. Larger distances could be found for profiles evoked by stimuli #1 and #9. Profiles evoked by the high frequency stimuli #3, #6, and #8 were found clustered together and distantly located from the remaining stimuli.

The two-dimensional configuration of Euclidean distances (stress = 0.003) calculated for the CSD data (Fig 5F) was qualitatively comparable to that found for LFP data. The CSD profiles evoked by stimuli #2, #4, and #5 clustered around stimulus #4 showing small distances, whereas the distances between profiles evoked by stimuli #1, #7, and #9 and the cluster consisting of stimuli #3, #6, and #8 were significantly larger. The MDS analysis thus reveals that different stimuli can be distinguished on the basis of stimulus-specific single layer activity waveforms. However, the stimulus discriminability is much better if whole laminar profiles are analyzed.

### 2.4. Degree of complexity in layer-specific waveforms and stimulus-specific profiles

Overall, the relatively large Euclidean distances between LFP and CSD profiles obtained with different stimuli rendered the LFP and CSD signals more suitable for stimulus-specific segregation (see Fig 5). The better segregation performance of LFP and CSD signals could originate from their higher structural complexity. To assess which of the three signal types (MUA, LFP, or CSD) bear the highest structural complexity we used the Kolmogorov complexity algorithm. This algorithm uses binary data to describe signal compressibility [57]. Repetitive sequences are compressed to a higher degree and lead to a lower bit size. The binary transformation border was set at 0 for LFP and CSD waveforms/profiles and at two times the individual median for MUA waveforms/profiles. Waveforms and profiles were individually normalized. The MUA waveforms and profiles were interpolated to match the number of points of LFP and CSD profiles (1 or 9 layers/sub-layers x 3000 time points, 20 kHz sampling rate).

**Fig 6:**
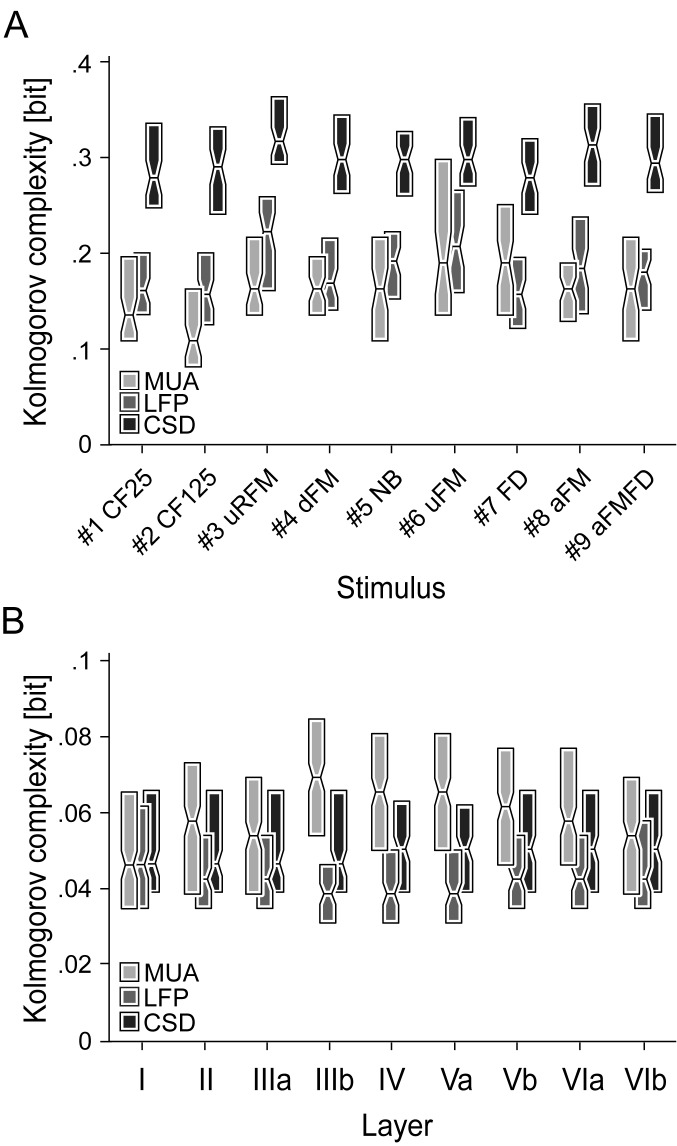
Kolmogorov complexity of layer-specific waveforms and laminar LFP, CSD, and MUA profiles. The Kolmogorov complexity indicating the compressibility of binary data is shown as color-coded boxplots for each stimulus (A) or layer (B). In (B) the responses to all stimuli at each layer were pooled together. Edge of box represents second and fourth quartiles and midline represents median of data. Binary transformation border was set at 0 for LFP and CSD waveforms and at two times the individual median for MUA waveforms. The stimulus-specific Kolmogorov complexity is highest for CSD profiles indicating a high structural complexity. The expectedly overall lower Kolmogorov complexity for stimulus-unspecific single layer waveforms is comparable in layers I-IIIa, but ranks as MUA > CSD > LFP in the remaining layers IIIb-VIb.

The results of the Kolmogorov complexity analysis are shown for the stimulus-specific laminar profiles (Fig 6A) and stimulus-unspecific single-layer waveforms (Fig 6B). Note that in Fig 6B, responses to all stimuli at each layer were pooled together. At the level of stimulus-specific laminar profiles, single LFP and MUA profiles showed values around 0.18 bits, while, on the other hand, the CSD profiles showed much higher values (mean = 0.3 bits) implying a higher structural complexity in the latter. This is not surprising, as CSD profiles are the 2nd spatial derivative of the LFPs and thus show considerably higher laminar variability and specificity than the volume conducted LFP activity. Interestingly, LFP and CSD profiles elicited by stimuli #3, #6, #8, and #9, showing high dissimilarities based on the large MDS distances (see Fig 5E and 5F), also possessed the highest complexity values, respectively. The Kolmogorov complexity was, as expected, lower at the single-layer level (Fig 6B), showing comparable values in layers I-IIIa for all three signal types (~0.05 bits). In the remaining layers (IIIb-VIb), complexity for the three signal types studied ranked as: MUA > CSD > LFP.

The high values found at the level of laminar profiles regarding the CSD data indicate a correlation between structural complexity and segregation performance. However, this correlation could not be found at the single layer level.

## Discussion

In this article, we describe MUA, LFP, and CSD responses evoked by complex communication sounds and simple pure-tones at the laminar and columnar level. We show that single-layer LFP and CSD activity characteristically depends on the acoustic stimulus type, in contrast to the MUA, which shows a lower “call-specificity”. The stimulus-specific segregation performance increases significantly (1.8-2.6 times) if whole laminar profiles including all cortical layers are analyzed, instead of single layer profiles. By directly comparing MUA, LFP, and CSD profiles we could show that the LFP and CSD recordings of single columns provide valuable information that could help distinguishing between different spectro-temporal call properties. The increased segregation performance which correlates with a higher structural complexity (only CSD profiles) and the high “call-specificity” in LFP and CSD profiles (higher than that in the MUA) reflecting population synaptic potentials supports the hypothesis that encoding of communication sounds depends not on single neurons but rather on population activity.

### 3.1. Anesthesia

The data reported in this manuscript was obtained from ketamine-anesthetized animals. To better understand the reported data, possible advantages and disadvantages of studying neural signals under the influence of ketamine need to be addressed. LFPs are influenced by nonspecific factors like ongoing cortical activity (spontaneous or evoked by a preceding stimulus) or the state of excitability [58], which can depend on a number of factors including attentional states [59] and/or context [60–61]. Complex processes such as attention are less likely to influence brain activity in anesthetized animals when compared to awake animals. However, anesthesia has complex effects on cerebral processing and previous studies have reported different impacts of anesthetics on MUA, LFP, electroencephalography or auditory brain-stem potentials [62]. Several anesthetics (pentobarbital, pentobarbital/ketamine, pentobarbital/chloral hydrate, droperidol, barbiturate, benzodiazepine, etomidate, propofol, and althesin) are known to impede neural conduction and synaptic transmission, thereby decreasing the amplitude and increasing the latency of evoked responses [62–66]. However, ketamine alone (one of the two pharmacological agents used in this study) has been associated with an amplitude and latency increase of cortical field potentials and auditory brain-stem evoked potentials at high doses [67–68]. A mixture of ketamine and xylazine did not alter the neuronal activity along the early stages (from brain-stem to inferior colliculus) of the ascending auditory pathway [69–71].

Studies regarding the influence of ketamine and xylazine on the complexity of recordings evoked by complex communication sounds are, at present, not available. Studying sound processing under anaesthesia could be useful because it describes the hardwired brain activity that is obtained without considering the influence of active processes such as attention. Such active processes could largely influence the sound discrimination based on activity from single layer and whole laminar profiles.

### 3.2. Segregation characteristics of single-layer waveforms and laminar MUA, LFP, and CSD profiles

Based on the Euclidean distances in the single-waveform MDS, the MUAs were less suited than LFPs and CSDs for sound discriminability, which is in accordance with previous studies regarding the MUAs and LFPs [12]. However, this is in contradiction to the results of the Kolmogorov complexity indicating the compressibility of binary data (Fig 6B). Single-layer MUA waveforms showed, in comparison to the LFP and CSD waveforms, the highest complexity. These results could have been caused by the complex spiking patterns in the area “a2” of the laminar response patterns that occurred following the initial phasic response (Fig 3Aa–3Ai). A similar delayed spiking activity was also found by Wallace and Palmer [72] in guinea pig responses to complex vocalizations. The similarities in the Euclidean distances of LFP waveforms and the layer-wise conformity in the MDS configuration (Fig 5B) is probably owed to the volume conduction [73] which is virtually eliminated in the layer-specific CSD waveforms as part of the calculation process [73].

Single-layer waveforms can be used for distinguishing different call properties at a decent level although they cannot provide all information about the sub- and intracortical processes induced by calls with complex spectro-temporal structures. Laminar profiles including all layers increased the segregation performance by factor 1.8-2.6 in comparison to the single-layer MDS (Fig 5). The LFP profiles showed the largest distances in the MDS ordering the segregation performance power as following: LFP > CSD > MUA. Medvedev and Kanwal [12] had already observed that MUA activity is less informative than LFPs and assumed that the MUAs were more sensitive to similarities present within the calls, whereas the LFPs were more sensitive to their differences, which is supported by the relatively large distances between LFP responses in the MDS (Fig 5E).

In contradiction to the hypothesis of Medvedev and Kanwal [12], in previous studies from Steinschneider and colleagues [5–6], structurally different consonant-vowel syllables elicited significantly different spike temporal patterns in cortical neurons of awake monkeys. Gehr and colleagues [11] could show the inefficiency, but not the impossibility, of a firing rate-based segregation between alterations of vocalizations in the AC. The MUA data described in this study is consistent with data described by Gehr and colleagues, since we show that MUA activity renders relatively weak segregation of sound-triggered activity in the MDS (see Fig 5D), at least when compared to the segregation produced by LFP and CSD waveforms. Of course, we cannot rule out the possibility that the observed spike segregation performance is sufficient for a proper categorization of sounds. Moreover, we cannot discard that the spike segregation performance could increase if the animals are awake (as opposed to anesthetized, as in this study), and actively trying to differentiate between sounds.

LFP waveforms, although appearing to be better suited for communication sound segregation, possess apparent similarities between cortical layers [74] and cannot provide information about the particular afferent inputs or underlying intrinsic synaptic processes that produce the laminar differences [41, 72].

Our data shows that the CSD profiles present a much higher structural complexity when compared to the MUA and LFP profiles (Fig 6A). Thus, it is suggested that laminar CSD profiles should be preferred for studying cortical activity. Of course, one should be aware of the different origins of LFP, CSD and MUA signals. While MUA signals reflect the neuronal output, CSDs and LFPs are likely to be influenced by ongoing synaptic potentials that do not necessarily lead to spiking, as well as by activity in extra-cortical areas and spike-bleed-through (i.e. the slow components of the action potentials that are kept in the LFP signals after filtering) [41–42].

### 3.3. Spectro-temporal call determinants for neural response patterns

The spectro-temporal cues of communication sounds, which are important for inducing characteristic activity patterns in the AC, are not yet fully understood. Medvedev and Kanwal [12] could show a correlation between call and LFP negativity duration, although LFP duration varied significantly within groups of calls of similar duration. In accordance to the latter, the present study could show that LFP and CSD profiles evoked by pure-tone stimuli of 25 ms or 125 ms duration differed significantly in the amplitude of N3 (LFP) and the sink s8 (CSD). Medvedev and Kanwal [12] hypothesized that the “harmonic complexity”, characterized as changes in the acoustic structure [predominant (peak) frequency, fundamental (lowest harmonic) frequency, and number of harmonics] is crucial for a differentiated temporal structure of the call-evoked LFPs. The harmonic structure of sounds can operate as an important information-bearing parameter for perception of call types and help in the detection of fine changes in the pitch [75]. Fishman and colleagues [9] could show that neural populations in the AI are able to resolve individual harmonics. Medvedev and Kanwal [12] assumed one of the dimensions in the MDS analysis of LFP waveforms to be correlated with the “harmonic complexity” and the other one with the predominant or fundamental frequency. This is in accordance with results of the present study, as the LFP and CSD profiles elicited by high frequency stimuli #3, #6, and #8, all possessing a low degree of “harmonic complexity” (see Fig 1), were located at the upper right part of the MDS plots. On the other hand, the LFP and CSD profiles that were evoked by low frequency stimuli, with a complex harmonic structure (#4, #5, and #7), were located at the lower left part of the MDS graphs (Fig 5E and 5F).

The spectral information may be less important as even severe loss (artificially-induced) does not prevent AC neurons from correctly classifying individual calls based on the MUAs [40]. In our data, the number of harmonics and the high central frequency in the three calls (#3, #6, and #8) eliciting strongly separated CSD and LFP profiles, were similar, but the calls differed in their FM slope, bandwidth, amplitude, and FM direction (see Fig 1), which have been described as important determinants for neural responses in the central auditory system [76].

### 3.4. Population coding of communication calls

Several studies have proposed that patterns of synchronized activity within cortical columns represent the acoustic attributes of different vocalizations [4, 7, 40] and may be well suited for the temporal encoding of pitch [9, 77]. Single cortical columns, which capture the activity of local cell assemblies and whose neurons respond similarly to the heard sounds since they are interconnected and tuned to similar frequencies [78–80], may be less suited for resolving the classification of calls, as they would lead to similar stimulus-induced activity waveform representations across layers. However, in the present study LFP and CSD waveforms recorded from neurons in the AI did clearly show layer-specific differences in the activity waveforms.

The LFPs in AI already represent the summed event-driven activity from a wide range of specific cortical loci involving the synaptic activity of several cell assemblies [41, 81] and do reflect the observed cortical spiking [15]. The increased structural complexity (Fig 6A) in the LFP and CSD profiles elicited by stimuli #3, #6, #8, and #9 showing high dissimilarities in the MDS (Fig 5E and 5F) suggests that the processing of specific stimuli involves additional activity of excitatory and/or inhibitory subsets of neurons in cortical and/or extra-cortical areas. This strengthens the model for encoding complex sounds based on activity patterns of neuronal populations spread throughout the cortex [4, 7, 40].

### 3.5. Possible origins of call-induced differences in the layer-specific activity

Based on the significantly different activity areas in the CSD profiles, obtained when comparing against the response to a control pure tone, the layers whose stimulus-dependent neuronal activity was responsible for most of the characteristic differences could be enclosed to granular (layer IV) and infragranular layers (layers V-VI). This was more obvious in response profiles showing less areas being significantly different to the reference response (Fig 4Ca, 4Cd and 4Cf).

The granular layer, which receives inputs from the ventral part of the medial geniculate body [52, 82] and the contralateral hemisphere, plays an important role in the intracortical processing [50]. The main output layers V and VI, besides receiving inputs from the medial part of the medial geniculate body, project to the thalamus and the inferior colliculus and receive inputs from higher areas like the enthorinal and frontal cortex [50, 83]. Both cortices play an important role in spatial memory, navigation, and cognitive control [84–87].

The synaptic activity associated to initial sinks s1 (layer IIIb/IV) and s2 (layer Vb/VIa), which are believed to reflect depolarizations of terminal portions of thalamic afferents and stellate cells [49, 88], showed stimulus-specific differences in their strength but not in their duration. This could suggest an activity-strength based coding of communication sounds in thalamic neurons. The mid and late evoked sinks in layers III/IV (s1b, s8), V (s5 and s9), and VI (s3, s3b, s6, and s7) were responsible for most of the structural differences in form of an absence of sinks (s7), a presence of sinks (s1b, s3b, and s9), and a temporal shift in sink location (s3 and s8). Their origins are still not fully understood, as they possess a relatively long cortical processing time during which many possible mechanisms could operate. It was speculated that late sinks might originate from repetitive thalamic after-discharges, intracortical processing mechanisms, or inputs from outside of the AC such as the frontal cortex, the contralateral hemisphere or the hippocampus [45, 89–91]. The sink s1b probably originates from thalamic after-discharges, as indicated by its laminar location and duration. The significant stimulus-specific differences in the late sink s8 could have been caused by intracolumnar processes, an altered synaptic activity in the contralateral hemisphere, or by feedback projections of higher cortical areas like the frontal cortex.

## Conclusions

The call-induced response differences, especially in the mid- and late-synaptic activity within cortical columns, suggest that not only thalamic and intracortical processes but also contralateral and/or higher cortical areas are involved in the call-specific processing that takes place at the cortical level. The structural differences in the laminar CSD profiles in response to calls produced by the presence and absence of sinks and differences in strength, latency, and duration allow a high-performance response profile-based discrimination for most of the sounds as shown using multi-dimensional scaling (Fig 5). The delayed infragranular sinks, located outside of the thalamo-recipient layers, could provide additional evidence for the hypothesis of the population coding of calls, as these sinks could directly or indirectly originate from population activity influenced by feedback projections of other cortical areas.

## Materials and methods

Experiments were conducted in 11 adult (age: 6-10 months; body weight: 44-79 g) Mongolian gerbils (*Meriones unguiculatus*) of both sexes (5 females and 6 males). Animals were taken from the breeding colony of the Institute for Cell Biology and Neuroscience, Goethe University, Frankfurt am Main, Germany. Animals were kept in polycarbonate cages (separated sexes) that were filled with nesting material (thickness: 3 to 6 cm). The breeding chamber was constantly kept at 21–22°C and under a 12:12 hours day-night-rhythm. Dry food and water was provided ad libitum. The study was in accordance with international guidelines for animal experimentation (International National Institutes of Health Guidelines for Animals in Research) and with ethical standards for the care and use of animals in research defined by German Law for the protection of experimental animals (Experimental permit: #F104/60). Ethics and animal experiments with ketamine/xylazine anesthesia was approved by the Regierungspräsidium Gießen (Approve number: #F104/60).

### 5.1. Sound recording

Communication sounds were recorded from animals of the same breeding colony. Apart from the downward frequency modulated sound (#4 dFM, Fig 1D), which was recorded from hand-held newborns, all sounds were recorded from adult animals. Two systems with different microphones were used to cover the emitted frequency range (~0.1-60 kHz). Most of the sounds (Fig 1C–1F and 1H) were recorded with a commercially available ultrasound recording system (UltraSoundGate 116Hm, Avisoft Bioacoustics, Berlin, Germany) and sound analysis system (Avisoft SASLab Pro, Version 5.1; Avisoft Bioacoustics) using a condenser microphone (UltraSoundGate CM16, Avisoft Bioacoustics) with a flat frequency response between 5-120 kHz. The two sounds containing low frequency foot-drumming (that is, #7 FD and #9 aFMFD, see Fig 1G and 1I) were recorded with a Brüel & Kjaer microphone (¼-inch Microphone 4135 and Microphone Preamplifier 2670, Brüel & Kjaer, Naerum, Denmark) connected to a Nexus amplifier (Nexus 2690, Brüel & Kjaer). The Brüel & Kjaer microphone had a flat frequency response between 0.004-100 kHz. For the audio recordings, the polycarbonate cage, where the animals were held, was placed in a soundproof room, and the microphone was attached 5 cm above the wire top of the cage. The bottom of the cage was covered with nesting material (3–6 cm thick). Recorded sounds were down-sampled to 192 kHz at 16 bits/sample.

Fig 1 shows two examples of artificial pure tones (#1 CF25 and #2 CF125, see Fig 1A and 1B) and the seven commonly emitted communication sounds which were selected to cover a variety of spectro-temporal properties and behavioral situations [92–93]. The intra-stimulus variety is relatively small for all in this study presented communication sounds with the exception of stimuli #4 and #5 [92–93]. The stimuli #4 and #5 utilized in this study capture the representative range of variety present in each communication sound, respectively. The upward ripple frequency modulated sound (#3 uRFM, Fig 1C) is known to be emitted by both adults and pups in mating and discomfort situations [92, 94]. The downward frequency modulated sound (#4 dFM, Fig 1D), recorded from hand-held newborns (1 day old), represents isolation calls, although these calls differ from classical isolation calls of gerbils recorded at day 3 or older [94–95]. The noise-burst (#5 NB, Fig 1E) is observed during agonistic interactions [92] whereas the upward frequency modulated sound (#6 uFM, Fig 1F) is known to be emitted during mating. The rhythmic foot-drumming of the rear limbs (#7 FD, Fig 1G) is commonly observed in combination with the arched frequency modulated sound (#8 aFM, Fig 1H) forming the arched frequency modulated + foot-drumming sound (#9 aFMFD, Fig 1I). This sound is probably unique among common laboratory rodents as it is a combination of vocal cord- and foot-produced sounds. The sounds #7-#9 are observed during alarm behavior [93]. Each communication sound consisted at least of two harmonics.

### 5.2. Acoustic stimulation

The individual communication sounds were cut out from longer sequences with a fade-in/out overhang of 10 ms to prevent clicks. Pure-tones were digitally synthesized and controlled using a custom-written program in MATLAB (R2007b, MathWorks, Natick, USA). The frequency of pure tones (duration: 25 or 125 ms; rise-fall: 5 ms) was set at the neuron's characteristic frequency (CF) obtained in the thalamic input layers V/VI. The CF (white asterisks in Fig 2A) was defined as the stimulus frequency that elicited a response at the minimum threshold (MT) in the frequency tuning curves. Two examples of tuning curves recorded from neurons tuned to low (left) and high frequencies (right) are displayed in Fig 2A. Tuning curves were calculated by presenting pseudorandomized series of pure tones at different levels (0-80 dB SPL) and frequencies (0.25-64 kHz). The threshold for calculating the receptive field was set at 30% of the maximum spike rate obtained in the tuning curve for each electrode channel. In the present study, only penetrations that yielded sensitive responses (MT < 50 dB SPL) were considered.

For further analysis of responses, pure-tones at the CF, along with the set of communication sounds described in the preceding text were played to the animals. The selected pure-tones were presented at 80 dB SPL (peak-to-peak amplitude). Communication sounds were attenuated by using the calibration curve and by taking into account the frequency with the highest power in the spectrum of each of the sounds [30.5 kHz (#3), 11.2 kHz (#4), 5.8 kHz (#5), 31.8 kHz (#6), 0.4 kHz (#7), 27.9 kHz (#8), 27.9 kHz (#9)]. The root mean square (RMS) values of the entire sound waveforms and the peak-to-peak (P2P) values are displayed in Table 1. The example pure-tones (#1 and #2) listed in the table were measured at a frequency of 2 kHz. The calibration curve for the used loudspeaker (SS-MS835, Sony, Tokyo, Japan), measured with a Brüel & Kjaer system (¼-inch Microphone 4135 and Microphone Preamplifier 2670, Brüel & Kjaer), was flat (± 9 dB) between 0.2-70 kHz. Sounds were generated via an external soundcard (e18 dac, exaSound, Toronto, Canada, sampling rate: 192 kHz), amplified (RB-1050, Rotel Electronics, Tokyo, Japan) and delivered through the loudspeaker. Communication sounds and chosen pure-tones were presented in a pseudorandomized order and repeated 50 times (delay: 50 ms; interstimulus interval: 600 ms). The speaker was placed in front of the animal’s right ear, at a distance of 20 cm.

**Table 1:** Root mean square (RMS) and peak-to-peak (P2P) values of the stimuli.

Stimulus	#1^a^	#2^a^	#3	#4	#5	#6	#7	#8	#9
RMS [dB SPL]	79.2	79.2	75.8	74.4	74.4	78.7	74.2	77.0	76.4
P2P [V]	0.95	0.99	1.11	0.79	0.89	1.04	0.74	0.96	0.95

^a^measured at 2 kHz

### 5.3. Anesthesia and surgical procedures

Initial anesthesia was given via intraperitoneal injection of a mixture of ketamine (100 mg/ml; Ketavet, Pfizer, New York, USA), xylazine (20 mg/ml; Rompun, 2%; BayerVital, Berlin, Germany) and isotonic sodium chloride solution (9 mg/ml; 0.9%; B. Braun, Melsungen, Germany). Continuous anesthesia was maintained with the same mixture with the aid of an injection pump (flow-rate: 0.75 mg/kg/h, Genie, Kent Scientific Corporation, Torrington, USA). The hind limb withdrawal reflex and whisker activity was monitored throughout the experiment. Body temperature was kept constant at 37°C using a thermostatic heating blanket.

For gaining access to the brain, the skin and muscle tissue of the upper and temporal part of the head was removed and the skull was cleaned. A custom-made metal rod (1 cm length, 0.3 cm diameter) was glued onto the skull using dental cement (Paladur; Heraeus Kulzer, Hanau, Germany). A square-shaped hole of ~3x3 mm was drilled into the temporal bone to expose the left auditory cortex (AC). After cleaning the brain surface, the *dura mater* was carefully removed with an injection needle. At the end of experiments, animals were euthanized with an overdose of pentobarbital (Narcoren, Merial GmbH, Hallbergmoos, Germany).

### 5.4. Electrophysiological recordings

Experiments were conducted in a custom-built soundproofed and electrically-shielded chamber. Recordings were made with commercially available linear probes (Model: A1x16-3mm-100-177-A16, NeuroNexus, Ann Arbor, USA) with 16 contacts (spacing: 100 μm) spanning 1500 μm. Due to the low impedance of the probes [0.5 to 3MΩ (as provided by the manufacturer)] the studied neuronal activity likely represents the summed activity of multiple nearby neurons, and it is therefore termed “multi-unit activity”. In the present manuscript, the term “neuron” is used to refer to multi-unit activity.

The orthogonality between the electrode and the brain surface was achieved by adjusting the animal's head several times until the altitude differences of the electrode tip at the four corners of the recording area was less than 60 μm [49]. This technique was applied once for each animal at the beginning of the experiment. Electrodes were inserted perpendicularly to the pial surface at a speed of 20 μm/s (micro-manipulator system: PM 10/1, Science Products GmbH, Hofheim, Germany) until the tip of the electrode reached a depth of 1500 μm. The 16 channels of the electrode covered all six cortical layers. The cortical layer separation was based on a previous study by Sugimoto et al. [79]: layer I: 0-120 μm; layer II: 120-210 μm; layer III: 210-410 μm; layer IV: 410-560 μm; layer V: 560-850 μm; layer VI: 850-1300 μm.

### 5.5. Recording site mapping

The cortical location of all 61 recording sites (sites per animal: 7, 5, 9, 3, 11, 5, 6, 5, 5, 1, and 4) studied were obtained by using the stereotaxic coordinates of penetration points and the suture intersection of parietal, sphenoidal and temporal bone as reference. Using these coordinates, the sites were plotted in the schematically parcellated AC [51–52] displayed next to the schematically illustrated skull in Fig 2B. The whole cortical map was constructed by overlaying the maps obtained in individual animals. The color-coded CFs reflect the tonotopic organization of the neurons in the AI and ranged between 0.5 and 32 kHz (Fig 2C). During the recordings, we tried to sample homogeneously from the entire surface of the AI to obtain an evenly distributed representation of neurons tuned to different frequencies.

### 5.6. LFP and spike activity analysis

The neuronal activity was preamplified (10x, μPA16, Multichannel Systems, Reutlingen, Germany) and recorded using a multichannel recording system (amplification: 1000x, ME32, Multichannel Systems). The raw signal, which was down-sampled from 50 to 20 kHz, was digitally bandpass-filtered offline (butterworth, 2^nd^ order) between 300 and 4500 Hz to obtain the MUAs (Fig 2D), and between 0.2 and 300 Hz for LFPs (Fig 2E). MUA profiles are generally composed of an initial activity (a1) and a delayed activity (a2), colored yellowish to red. LFP profiles generally consist of two initial negativities (N1 and N1b) and a delayed negativity (N3). LFP waveforms were additionally notch-filtered at 50 Hz to remove power-line noise. Peri-stimulus time histograms (PSTHs, 2 ms bin size) were calculated from the time of fired spikes. The threshold for spike detection was set to 2.5 times the standard deviation of the baseline noise (50 repetitions of acoustic stimuli) using a dead-time of 1 ms. Throughout the manuscript, we will refer to the MUA-based PSTH as “MUA waveforms”.

### 5.7. Current source density analysis

The standard CSD method assumes a homogeneous activity along the horizontal direction. It uses a discretized version of the Poisson’s equation and assumes the extracellular medium to act as a volume conductor that is ohmic at the relevant frequency range [44–45, 96–97]. The one-dimensional CSD profiles, which are approximations of the real current flows, were calculated from the second spatial derivative of the LFPs averaged over 50 stimulus repetitions using the following formula:

$$\frac{\delta^{2}\phi }{\delta z^{2}}\approx \frac{\phi (z_{0}+n\Delta z)+\phi (z_{0}-n\Delta z)-2\phi (z_{0})}{{\left(n\Delta z\right)}^{2}}$$

The double of the field potential (ϕ) at the cortical depth z_0_ is subtracted from the summated adjacent field potentials above (z_0_+nΔz) and below (z_0_-nΔz) the field potentials at depth z_0_ (interchannel distance Δz = 100 μm) and divided by the differentiation grid (nΔz; n = 1). A modified version of the iCSDplotter toolbox was used for the CSD calculation [97]. The top and bottom electrode channels were estimated with the method of Vaknin et al. [98]. To reduce spatial noise, a three-point Hamming filter was applied [99]:

$$\phi_{\mathrm{filt}}(z)=0.23\phi (z_{0}+n\Delta z)+0.23\phi (z_{0}-n\Delta z)+0.54\phi (z_{0})$$

The current sinks (colored in red) in the laminar CSD profiles (Fig 2F) are classically interpreted to indicate excitatory events, e.g. axonal depolarizations and excitatory or inhibitory synaptic activations, while current sources are supposed to represent in most cases passive return currents [45]. The basic CSD profile within a 600 ms window exhibits two initial sinks (s1 and s2) and 6 secondary sinks (s3-s8). Visualization of laminar profiles was improved by linear channel interpolation. All area contours (MUA, LFP, and CSD) were calculated using MATLAB's “*contour*” function.

### 5.8. Statistical analysis

Normal distribution was verified with the Lilliefors-Test using the MATLAB function “*lillietest*”. A parametric repeated measures analysis of variance (Salarian, Arash (2008). “*anova_rm*”, MATLAB Central File Exchange, Retrieved January 9, 2016) was then applied in combination with a false discovery rate post-hoc test [53] using the MATLAB function “*mafdr*”. Tests that rendered p values < 0.05 were considered as significant.

## Supporting information

S1 Fig Interquartile range of MUA, LFP and CSD profiles of stimulus-evoked recordings. Each point in the profiles represents the interquartile range of the depth-adjusted (n = 61) MUA (Aa-Ai), LFP (Ba-Bi), and CSD (Ca-Ci) in the response to two pure tones (rows 1-2) and 7 different complex sounds (rows 3-9). Interquartile ranges of LFPs and CSDs were exclusively calculated for the negativities (Ba-Bi) and Sinks (Ca-Ci). The neuronal activity [a1-a4 (MUA), N1-N3 (LFP), and s1-s9 (CSD)] is marked at the same spot as in Fig 3. The dispersion of median profiles within the same stimulus group remains overall relatively low and is slightly enhanced in the initial activity areas (a1, N1, s1, and s2) and in some secondary areas (N3, s3, s4, and s8) while ranging within the stimulus-specific profile structure. This indicates that the median profiles represent the stimulus-specific structure found at the level of single laminar profiles. The vertical dashed line marks the beginning of stimulation. Corresponding oscillograms of stimuli are shown above the profiles.
(TIF)

S2 Fig Representative MUA, LFP, and CSD responses of layer IV neurons tuned to low and high frequencies. The different neuronal-specific receptive fields across the mapped cortical region (CF < 6 kHz: n = 32; CF > 6 kHz: n = 29, see Fig 2C) could affect the stimulus response. In order to observe receptive field specific changes the MUA-based peri-stimulus time histogram (in short: MUA; upper row), LFP (middle row), and CSD responses (lower row) of a low frequency (black) and a high frequency tuned (gray) cortical site of the same animal are shown in each sub-panel. The neuronal tuning curves of both cortical sites are displayed in Fig 2A. Stimuli whose frequency content was limited either to high (#3, #6, and #8) or to low frequencies (#7) result in strongly decreased MUAs in neurons tuned to frequencies outside of the stimulus frequency range. The additional small spiking activity appearing at the time of the second drumming event of stimulus #7 can be seen in the enlarged view. Different neuronal-specific receptive fields do elicit similar stimulus-specific waveforms. However, LFP and CSD waveforms obtained at both cortical sites were much more similar than the MUA waveforms. The oscillograms of stimuli are shown below the responses at the same time scale.
(EPS)

S3 Fig Correlation coefficients between waveforms recorded from low and high frequency tuned neurons. The figure shows the correlation coefficients (zero time lag) of single MUA, LFP, and CSD waveforms (averaged over 50 trials) between low frequency (n = 32) and high frequency tuned neurons (n = 29) of layer IV. Each waveform of one of the two frequency groups was correlated with all the waveforms of the opposite frequency group. Boxplot data is composed of the highest correlation coefficients between the three channels spanning layer IV of each measured stimuli, respectively. The edge of box represents the second and fourth quartiles and the midline represents the median of data. Lowest correlation coefficients (> 0.27) are found in MUA waveforms evoked by stimuli exclusively consisting of high frequencies (#3, #6, and #8). These frequency-dependent dissimilarities are mainly related to the spiking activity strength. The overall high correlation coefficients indicate that different receptive fields do not alter the stimulus-specific processing patterns of waveforms in a fundamental way.
(EPS)

S4 Fig Repeated measures ANOVA between all cross-compared laminar MUA, LFP, and CSD profiles. A parametric repeated measures ANOVA in combination with a false discovery rate post-hoc test was applied for 61 measurement points at each of the 600 time points and all 16 channels of the MUAs (A); the LFPs (B), and the CSDs (C). Each stimulus-evoked profile was tested against each other with the exception of the profile evoked by stimulus #2 CF125 which is already displayed in Fig 4. The points in the profiles represent the probability values (green: p ≥ 0.05; yellow: p < 0.05; red: p < 0.01; dark red: p < 0.001). Horizontal dashed lines indicate layer borders while the vertical dashed line marks the beginning of stimulation. Sounds differing in their spectro-temporal properties evoke significantly altered MUA, LFP, and CSD profiles. MUA profiles show the fewest significantly altered locations in comparison to the LFP and CSD profiles. However, in relative terms profiles that do show a high amount of significantly altered locations are the same in all three signal types.
(EPS)

## Abbreviations

AC: auditory cortex
aFM: arched frequency modulated
aFMFD: arched frequency modulated foot-drumming
AI: primary auditory cortex
CF: characteristic frequency
CSD: current source density
dFM: downward frequency modulated
FD: foot-drumming
FM: frequency-modulated
LFP: local field potential
MDS: multi-dimensional scaling
MS: magnitude of significance index
MT: minimum threshold
MUA: multi-unit activity
NB: noise-burst
P2P: peak-to-peak
PSTH: peri-stimulus time histogram
RMS: root mean square
SD: standard deviation
uFM: upward frequency modulated
uRFM: upward ripple frequency modulated.
