# Supplementary figures and images for: Laminar differences in response to simple and spectro-temporally complex sounds in the primary auditory cortex of ketamine-anesthetized gerbils
Source: PLoS One. 2017 Aug 3;12(8):e0182514. doi: 10.1371/journal.pone.0182514 (PMC5542772; doi:10.1371/journal.pone.0182514)

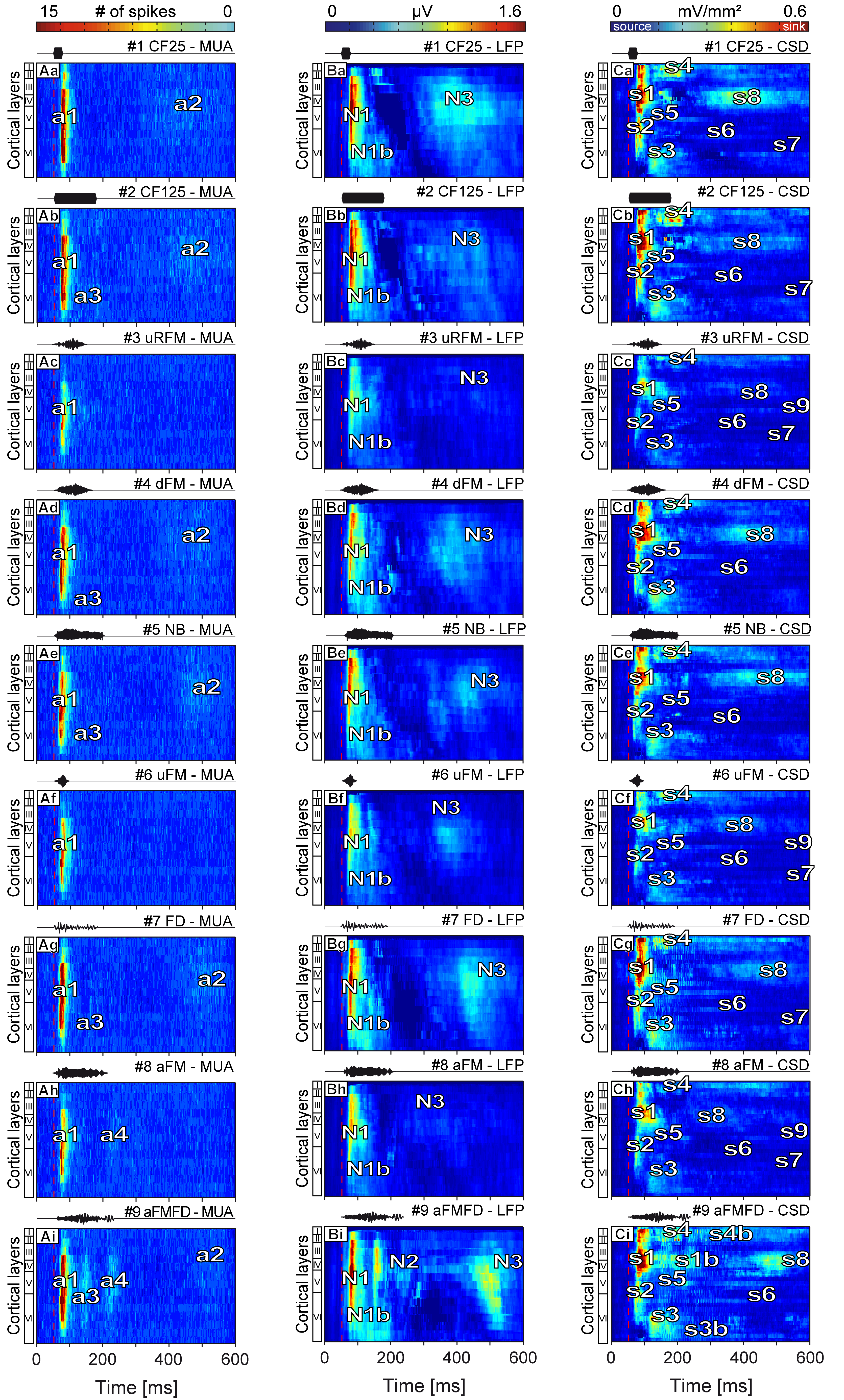

Supplement: S1 Fig — Each point in the profiles represents the interquartile range of the depth-adjusted (n = 61) MUA (Aa-Ai), LFP (Ba-Bi), and CSD (Ca-Ci) in the response to two pure tones (rows 1-2) and 7 different complex sounds (rows 3-9). Interquartile ranges of LFPs and CSDs were exclusively calculated for the negativities (Ba-Bi) and Sinks (Ca-Ci). The neuronal activity [a1-a4 (MUA), N1-N3 (LFP), and s1-s9 (CSD)] is marked at the same spot as in Fig 3. The dispersion of median profiles within the same stimulus group remains overall relatively low and is slightly enhanced in the initial activity areas (a1, N1, s1, and s2) and in some secondary areas (N3, s3, s4, and s8) while ranging within the stimulus-specific profile structure. This indicates that the median profiles represent the stimulus-specific structure found at the level of single laminar profiles. The vertical dashed line marks the beginning of stimulation. Corresponding oscillograms of stimuli are shown above the profiles. (TIF) [file pone.0182514.s001.tif]
